# What Do We Know of Childhood Exposures to Metals (Arsenic, Cadmium, Lead, and Mercury) in Emerging Market Countries?

**DOI:** 10.1155/2013/872596

**Published:** 2013-01-08

**Authors:** Lindsey M. Horton, Mary E. Mortensen, Yulia Iossifova, Marlena M. Wald, Paula Burgess

**Affiliations:** ^1^Office of Science, National Center for Environmental Health and Agency for Toxic Substances and Disease Registry, 4770 Buford Highway, Atlanta, GA 30341, USA; ^2^Division of Laboratory Sciences, National Center for Environmental Health, Centers for Disease Control and Prevention, 4770 Buford Highway, Atlanta, GA 30341, USA

## Abstract

Arsenic, cadmium, lead, and mercury present potential health risks to children who are exposed through inhalation or ingestion. Emerging Market countries experience rapid industrial development that may coincide with the increased release of these metals into the environment. A literature review was conducted for English language articles from the 21st century on pediatric exposures to arsenic, cadmium, lead, and mercury in the International Monetary Fund's (IMF) top 10 Emerging Market countries: Brazil, China, India, Indonesia, Mexico, Poland, Russia, South Korea, Taiwan, and Turkey. Seventy-six peer-reviewed, published studies on pediatric exposure to metals met the inclusion criteria. The reported concentrations of metals in blood and urine from these studies were generally higher than US reference values, and many studies identified adverse health effects associated with metals exposure. Evidence of exposure to metals in the pediatric population of these Emerging Market countries demonstrates a need for interventions to reduce exposure and efforts to establish country-specific reference values through surveillance or biomonitoring. The findings from review of these 10 countries also suggest the need for country-specific public health policies and clinician education in Emerging Markets.

## 1. Introduction

Arsenic, cadmium, lead, and mercury have been studied extensively due to the known serious adverse health effects associated with human exposure to these metals [1–4]. Although arsenic is a metalloid, it is commonly referred to as a metal; for the purposes of this paper, the term "metal" is used for arsenic, cadmium, lead, and mercury. Anthropogenic sources of these metals in the environment worldwide include industrial emissions, fossil fuel burning, waste incineration, consumer products, and mining and smelting wastes [5, 6]. With rapid economic development and limited regulatory infrastructure to provide oversight, developing countries provide instances of large scale and cottage industries releasing metals into the environment [5, 7–9].

Human exposure to arsenic, cadmium, lead, and mercury is primarily a result of inhalation of metal particles in air, ingestion of contaminated food or drinking water, or ingestion as a result of hand-to-mouth behavior [10–13]. Fetal exposure occurs when metals cross the placental barrier, and infants may also be exposed to arsenic, cadmium, lead, and mercury through breastfeeding. Significant inorganic arsenic exposure occurs through the consumption of drinking water as a result of geologically contaminated groundwater sources in particular regions of the world [14–17]. Children may also be exposed to arsenic by ingesting contaminated soils and dust or coming in contact with wood surfaces preserved with chromated copper arsenate [18, 19]. In addition, dietary sources of both arsenic and cadmium contribute to background levels of these metals in the general population, and, occasionally, these dietary sources also have become highly contaminated from pollution. Cadmium exposure occurs through inhalation or ingestion, with dietary sources contributing the majority of body burden for tobacco nonsmokers [20, 21]. Lead can enter the body when fine lead particulates are inhaled or lead compounds are ingested. Children are frequently exposed to lead when hand-to-mouth behaviors result in ingestion of lead-based paint and lead-contaminated dust [4, 22]. Prenatal and early childhood lead exposure is of particular concern because children absorb lead more readily than do adults, and lead has the ability to affect developing organ systems. All of the countries included in this paper have banned the use of leaded gasoline, an action that has been associated with a more than 90% decrease in blood lead levels as well as a 5-6 points increase in mean population IQ scores in the United States since 1976 [23]. Elemental mercury, which is used in artisanal gold mining, results in exposure through inhalation of the vapor. In the body, elemental mercury distributes to the brain and tissues, where it is converted to inorganic mercury [24, 25]. Discharged into freshwater streams and waterways, elemental and inorganic mercury can be methylated by microorganisms. The resulting methylmercury bioaccumulates in the food chain of freshwater streams and waterways; consequently, fish may have elevated methylmercury levels. Consumption of affected fish acts as a potential source of human exposure to mercury. Several predatory species of ocean fish which are higher in the food chain are known to have elevated methylmercury levels despite no obvious contamination source [26].

Human exposure to these four metals is best assessed by blood and/or urine measurements. Urine arsenic is a biomarker of recent exposure, and levels have been correlated with arsenic intake from drinking water and dietary sources [14, 15, 27]. Speciation of urine arsenic distinguishes the more toxic inorganic forms from the relatively nontoxic organic forms that derive from seafood consumption and may be referred to as “seafood arsenic” [28]. Blood cadmium reflects both recent and cumulative exposures. Urine cadmium reflects cumulative exposure as well as the concentration of cadmium in the kidney, which is the target for toxicity and the repository for one-third to one-half the body burden of cadmium [29, 30]. Whole blood lead measurement is the standard method to evaluate lead exposure and reflects both recent intake and equilibration with lead stored in other tissues, especially bone. Total blood mercury, often simply referred to as “blood mercury,” is mostly a measure of dietary intake of methylmercury and, in the absence of significant inorganic mercury exposure, is about 95% methylmercury and reflects the body burden [3]. In contrast to blood, urinary mercury consists of largely inorganic mercury [31, 32]. Hair and nails have been used to assess metals exposure, but, for the most part, these provide semiquantitative results, and specimen selection, preparation, removal of external contamination, and analysis are not wellstandardized. 

Children and infants may have higher exposure to metals because they consume more food in relation to their body weight and absorb metals more readily than adults [33]. Methylmercury and lead exposures during pregnancy and early childhood have adverse effects on the developing nervous system, and lead exposure during early childhood, even at low levels, has been associated with numerous neurodevelopmental effects including lower IQ, cognitive impairments, increased risk for attention deficit hyperactivity disorder, and impulsivity [4, 34, 35]. Prolonged exposure to arsenic beginning in childhood may increase the likelihood of skin and internal cancers that have a long latency period [36]. Health effects of cadmium exposure in children may include kidney, lung, and intestinal damage, and animal studies suggest that children are more susceptible than adults to bone demineralization and fractures as a result of cadmium exposure [11]. Low level exposures to the combination of arsenic, cadmium, lead, and mercury may cause subtle effects on children's renal and dopaminergic systems [37].

This paper focuses on arsenic, cadmium, lead, and mercury exposure to children in countries that make up the world's top 10 Emerging Markets as classified by the IMF: Brazil, China, India, Indonesia, Mexico, Poland, Russia, South Korea, Taiwan, and Turkey [106]. Emerging Markets are characterized by a transition from closed to open markets, increased foreign investment, and a shift from agriculture to industry, [107, 108] and they comprise approximately 80% of the world's population [109]. Two features common to all Emerging Market societies are rapid industrialization and increased urbanization [107], typically accompanied by pollution, environmental degradation, and industrial facilities built in close proximity to communities. The 21st century has seen increased globalization leading to the rise of Emerging Market countries as important participants in the global economy [108]. Authors conducted a literature review of 21st century English language articles on pediatric exposures to arsenic, cadmium, lead, and mercury in the IMF's top 10 Emerging Market countries where industrialization and urbanization may contribute to human exposure to metals. This literature review provides a general overview of pediatric exposure routes for common metals as well as blood and urine levels reported in studies of children in Emerging Market countries.

## 2. Materials and Methods

Structured database searches were conducted for published, peer-reviewed journal articles within the OVID versions of Medline and EmBase, as well as CAB Direct, for the years 2000–2012. Controlled vocabulary terms were identified in the thesaurus of each database and used consistently for search queries across all three databases. Authors selected search terms “blood” and “urine” to retrieve only articles that included an established measure of metal exposure. The terms used were “blood” and “urine” for matrix analyzed; “arsenic,” “cadmium,” “lead,” and “mercury” for metals of interest; “children” ≤ 18 years for our population of interest. These subject terms were combined with individual country names (Brazil, China, India, Indonesia, Mexico, Poland, Russia, South Korea, Taiwan, and Turkey), and retrieval was limited to English language articles. For the Medline and EmBase searches, authors integrated the additional controlled vocabulary term “exposure” into the search strategy to refine retrieval for populationbased studies with a public health focus. Searches were limited to articles that included blood or urine measurements in order to exclude non-standard matrices (e.g., hair and fingernails).  Table 1 shows the inclusion criteria for articles reviewed. 

Retrieved citations and abstracts were reviewed by the authors to identify any non-English language articles inadvertently retrieved for exclusion. A data extraction form was created to ensure that each reviewer could record specific data including metal(s) of interest, study objective, analytical method used, evidence of contamination, adverse health effect(s), and impact of results. Review articles, poster presentations, and abstracts were excluded, as was one article that focused solely on dental amalgam fillings, because this was not considered to be an ambient environmental exposure source. A total of 130 articles were read, and 76 met inclusion criteria. These articles represent the results of a structured, targeted database search; individuals could expand this search to find additional articles by adding additional search terms. A second level of review was provided by an author who confirmed completeness of biological results and sources that were abstracted.

## 3. Results and Discussion

### 3.1. Overview of Articles Reviewed

Of the 76 articles, one reported data from Russia, two each from Indonesia, Republic Korea, and Turkey, five from Brazil, six from Taiwan, nine from Poland, 12 from India, 18 from China, and 20 from Mexico. Because authors anticipated that a larger number of published articles would meet the inclusion criteria, additional searches were conducted to retrieve non-English language articles in MedLine, EmBase, and CAB Direct as a comparison. A total of 24 additional articles were identified that fit the remaining inclusion criteria, bringing the total to 100 peer-reviewed, published journal articles on this topic. The 24 non-English language articles were not formally translated or reviewed as part of this paper.

Of the 76 English language studies reviewed, 58 (76%) were conducted to inform public health (e.g., assessment of exposures and health effects, surveillance, evaluation of the effects of public health interventions). The remaining 18 (24%) were conducted to the further understanding of basic science concepts (e.g., interactions with physiologic, metabolic, or genetic processes) or evaluate therapeutic interventions. Most manuscripts identified by this literature review were published in journals based in developed countries and authored by academic researchers. Many of the studies were conducted by investigators from non-Emerging Market countries and/or funded by United States (US) and United Nations sources. Lead was the most commonly studied metal, and 55 articles focused on lead or a combination of lead and other metals. Because developed countries such as the US and countries in the European Union have dedicated substantial and largely successful efforts to reducing lead exposure, it might be expected that there are more pediatric lead studies in Emerging Markets than studies of other metals.

A large number of studies focused on newborns and infants, with 32 (42%) reporting metal concentrations in cord blood. Study populations in the remaining 44 (58%) articles ranged from ages 1 to 18 years. In only four studies (5%) was the sample size more than 1000 children, and in 22 studies (29%), the samples were less than 100. Many of the smaller studies were investigations conducted near sites where metal exposure was documented or suspected as a result of industrial or mining-related activities. The study design for the majority of articles was cross-sectional cohort, although several reported blood lead measurements over multiple years. Five studies reported results for exposed and unexposed control groups.

### 3.2. Sources of Metal Exposure

The majority of studies described environmental sources of metal exposure, with many reporting high blood or urine levels as a consequence of metal contamination from nearby industrial activities. Two articles described occupational exposures in children and adolescents [95, 102]. One of these described mercury exposure from gold mining in Indonesia [95], and one described blood lead levels in teenagers employed in an auto repair business in Turkey [102]. Two studies described occupational take-home exposures of lead in children living with parents who were employed in mining and smelting industries [64, 89]. Worker education and improved industrial hygiene practices are well-known interventions that could be implemented and have been effective in reducing occupational take-home exposures in developed countries. 

A variety of industries were reported as known or suspected contamination sources in the 76 papers reviewed. Mining and smelting activities were the most frequently identified sources of metal release to the environment. Other industries included electronic waste recycling, automobile parts manufacturing, textile production, and general industrial activities. Coal-burning stoves were the primary source for metal contamination reported in several studies. Other articles identified past use of leaded gasoline and urban vehicle pollution as primary sources of environmental lead exposure. Deposits and runoff from natural geologic formations were the sources of arsenic in drinking water affecting very large populations in studies of arsenic exposure conducted in India, Mexico, and Brazil. Three articles reported exposure to lead from paint or ceramic pottery [66, 82, 96], and one described increased blood lead levels in Indian children due to the use of traditional cosmetics and powders containing lead sulfide [69].

### 3.3. Indications of Exposure

Table 2 summarizes blood and/or urine results for 69 of the 76 studies reviewed in order to show the type of results obtained from a variety of different study designs (e.g., cross-sectional cohort, case-control, and convenience sample) in several countries. Seven studies were excluded from the table because they combined blood and/or urine results for pediatric and adult subjects or did not report values of metals in blood and/or urine [111–117]. Studies that did describe the blood and/or urine analyses used standard analytical methods (e.g., ICP-MS, graphite furnace AAS) but frequently did not report limits of detection, detection frequency, or statistical handling of nondetectable values. Urine results were reported as either metal concentration in mass units or as creatinine corrected. The majority of articles reviewed did not include statistical analysis other than descriptive statistics, and those that did were small and underpowered. Summary statistics were also varied: geometric means, arithmetic means, medians, or ranges of values. These differences limited comparisons among the studies and with established reference values. Authors chose not to present *P* values or confidence intervals for the few studies that included them due to the potential for overinterpreting study results. Table 2 is therefore purely descriptive and is not designed to present the detailed information that might be included in a traditional review or meta-analysis. In general, country-specific reference ranges were not available, which presents challenges to interpreting study results. Because national biomonitoring is not conducted in these countries, it is difficult to know the background levels of metals for the general population and, therefore, whether levels reported in some of these studies are unusually high.

The Centers for Disease Control and Prevention/Agency for Toxic Substances and Disease Registry (CDC/ATSDR) conduct biomonitoring using a representative sample of the US population that participates in the National Health and Nutrition Examination Survey (NHANES; additional details are available at http://www.cdc.gov/nchs/nhanes.htm). Urine metals and creatinine are measured in participants aged 6 years and older, and blood metals are measured in participants aged 1 year and older. Blood and urine analyses are conducted by CDC's Environmental Health Laboratory, and results are compiled and reported in the National Report on Human Exposure to Environmental Chemicals [110].  Table 3 presents US reference values (95th percentile estimates) by age groups when available for arsenic, cadmium, lead, and mercury using NHANES 2005-2006 results. This survey period was selected because it occurred approximately in the middle of the literature search timeframe, thus providing potentially relevant values for comparison. Of note, CDC has recently revised its recommendations regarding elevated blood lead levels in children. The previous guidance has been replaced with a reference value based on the 97.5th percentile of children aged 1–5 years old from the two most recent two-year NHANES survey periods; this value is currently 5 *μ*g/dL but could change in subsequent survey periods [118]. 

The majority of study results from Emerging Market countries reported values that were elevated relative to U.S. general population values from NHANES. This was even the case in the unexposed groups used in several small studies that compared exposed and relatively unexposed individuals. Any comparison between metals concentrations reported in the studies and the U.S. NHANES is limited however, because the U.S. data provides reference values that are representative of a country where environmental regulations are stricter, industry is often outsourced, local industrial facilities may be monitored for compliance, and there is greater awareness of environmental public health than in Emerging Market countries.

### 3.4. Health Effects Reported

The study designs, data analyses, and reporting of health outcomes varied greatly among studies and limited our ability to summarize health effects. Studies of childhood arsenic exposures reported significant associations between levels of arsenic in blood or urine and precancerous skin lesions. Three studies of Indian populations in regions with arsenic-contaminated drinking water included descriptions of children with evidence of health effects including characteristic arsenic-induced skin lesions and varying degrees of peripheral neuropathy [40, 41, 115]. Other studies revealed negative associations between levels of arsenic in blood or urine and birth weight, gestational age, children's cognitive test scores, and measures of IQ [39, 42, 44].

Seven articles described cadmium exposures in children. Three found negative associations between cord blood cadmium and birth outcomes (e.g., birth height birth weight) in infants [51, 52, 56]. A study of neonatal cadmium exposure in China reported low birth weight as well as slightly decreased IQ at age 4.5 years associated with higher levels of cord blood cadmium [51]. Another study from Taiwan found that cord blood cadmium was inversely associated with newborn head circumference, height, and weight up to age 3 years [56].

Fifty-five articles discussed lead exposure, and at least one study on lead was conducted in each of the top 10 Emerging Market countries. Findings were similar to those from studies conducted in the U.S. and other developed countries, with subtle but negative associations between blood lead levels and neurological, behavioral, and mental development test scores [60, 61, 76–79, 86, 87, 94]. In a Polish study of low-level prenatal lead exposure (median cord blood lead level = 1.23 *μ*g/dL), a significant deficit in Mental Development Index scores persisted at 1, 2, and 3 years of age [86]. Other health effects associated with lead exposure in these studies were low birth weight, aplastic anemia, and stunted growth [68, 71, 89].

Of the 12 mercury exposure studies, three reported associations between total blood mercury levels and lower mental and psychomotor developmental test scores [102, 105, 117]. A study of occupational mercury exposure in Indonesia found that exposed children between the ages of 9–17 years had a higher frequency of clinical signs of inorganic mercury toxicity including ataxia, dysdiadokinesis, and pathological reflexes than did their unexposed counterparts [102]. One study of prenatal exposure to methylmercury in Brazil found positive associations between maternal and cord blood mercury levels and IgG levels, suggesting that prenatal mercury exposure may have immunologic effects in the fetus [99].

### 3.5. Challenges

The majority of studies reviewed in this paper are investigations of chemical exposures and assessments of public health impact at contaminated sites. These studies require collection and analysis of site-specific environmental data, human biomarkers, and health outcome data to determine whether people have been exposed to hazardous substances that may cause negative health outcomes. There are a number of challenges associated with chemical exposure investigation and public health impact assessment that may contribute to our limitations in comparing biological sampling data and summarizing health effects. These studies are site-specific, and each site presents unique challenges in terms of evaluating exposure pathways and identifying the population at risk, contaminant source(s), affected media, and possible health effects in the population. As a result, scientists often have difficulty been showing associations with environmental contaminant exposures and adverse public health outcomes. Sites may have various information gaps that require additional sample collection and analysis; financial and time constraints may limit scientists in their ability to gather necessary data to properly evaluate site-related exposures. Even in cases where a scientist is able to gather necessary blood and/or urine samples for analyses, there may be missing information on contaminants in environmental media that create challenges in establishing possible associations. Health outcome data can be difficult to evaluate, as self-reporting on surveys and questionnaires can be biased and difficult to validate while medical records may be nonexistent or challenging to obtain as a result of confidentiality issues.

Carrying these investigations further through epidemiological studies to discern whether a particular exposure is associated with or causes a particular adverse health consequence presents other difficulties. Environmental epidemiological studies are methodologically complicated due to not only the challenge of identifying and quantitating chemical-specific exposures, but also because there may be multiple potential confounders, not all of which can be measured or identified. Obtaining adequate statistical power to draw meaningful conclusions depends on having an adequate sample size, which can be difficult in site-specific exposure scenarios. Even when the exposure to a specific chemical can be identified, potential adverse health effects can be measured, and the relevant biologic sampling and analysis can be performed on a relatively large number of individuals at a site; the epidemiologic analysis does not determine causation. Instead, environmental health epidemiological studies can at best identify statistically significant associations between exposure and outcome. 

Although standard analytical methods exist and are used for testing biological samples of arsenic, cadmium, lead, and mercury in the laboratory, authors identified variable approaches to data analysis and presentation of laboratory results in the studies published from Emerging Market countries. It is difficult to compare studies with one another and with established reference values when results are not reported consistently and statistical methods are varied. Site-specific characteristics including size and age of affected population, levels of chemical(s) or their metabolites detected in blood and/or urine, and detection limits for laboratory methods may also affect the reporting of results making any comparison between studies difficult. Even in the US, where population reference ranges exist for environmental contaminants, it may be difficult to determine the extent of exposure in specific subpopulations or in populations within particular geographic areas. Scientists in Emerging Markets face additional challenges when attempting to draw conclusions from their findings in countries where established reference ranges do not exist. 

In the US, ATSDR is the federal agency that investigates and seeks to prevent health effects related to human exposure to environmental hazards. Scientists at ATSDR use the Public Health Assessment Guidance Manual (available online at http://www.atsdr.cdc.gov/hac/phamanual/) when evaluating the potential for health impacts from environmental exposures to people at potentially contaminated sites [119]. This manual offers guidance on procedures for hazard identification, exposure investigation, data analysis, public health action, and community involvement when certain environmental contaminants have been discovered at a site. Although the process remains challenging and site-specific differences persist, ATSDR benefits from a standardized approach and consistent techniques for health assessment. Furthermore, ATSDR reports all results consistently, and this approach has been adopted by many public health agencies and organizations in the US, allowing for better comparability between sites and with established reference ranges. To our knowledge, there is no similar international guidance, and scientists in Emerging Market countries use a variety of approaches to evaluating environmental exposures and potential health effects to a community.

Conducting chemical exposure investigations, assessments of public health impact, or epidemiologic studies in Emerging Market countries can impose additional challenges beyond the inherent scientific ones. There can be infrastructure issues that limit the ability to conduct investigations. Technological issues may also affect the collection, transport, and storage of specimens to ensure that they remain free from contamination and can provide valid results. Developing countries face challenges in terms of the availability of expensive laboratory technology and staff trained and experienced in making quality measurements. The nature and extent of political issues and cultural sensitivities can add layers of complexity to the scientific undertakings as well. Finally, resources may be limited to interpret and provide results to study participants and work with communities to reduce exposures where appropriate. The work of scientists in Emerging Market countries is undoubtedly challenged by some or most of these issues. 

## 4. Conclusions

Arsenic, cadmium, lead, and mercury present potential health risks to children who are exposed at even low levels. Emerging Market countries experience rapid industrial development that may coincide with the increased release of these metals into the environment. In some regions of these countries, widespread contamination of drinking water and soils from naturally occurring arsenic may overwhelm available mitigation resources and technology. Authors' conclusions and recommendations based on the review of articles in this paper are summarized in Table 4.

The studies reviewed indicate evidence of pediatric exposure to arsenic, cadmium, lead, and mercury in Emerging Market countries. The reported blood and urine concentrations of metals in these studies were generally increased, relative to US reference values. Country-specific reference values are largely unavailable in Emerging Market countries making interpretation of exposure assessment difficult. Adverse health effects were reported that were likely a consequence of metals exposure in several of the Emerging Market countries, but there were challenges establishing causation and determining associations between exposure and health outcomes in the smaller studies.

In summary, the studies reviewed were conducted in 10 Emerging Market countries and reported pediatric metals exposures of concern related to specific industries and activities and to naturally occurring arsenic exposure. These exposures threaten the health of children as well as adults. Interventions to reduce exposures exist or can be designed and tested. To document progress in reducing exposure, evaluate interventions, identify specific at-risk subpopulations, and establish country-specific reference values, surveillance or biomonitoring could be implemented with technical assistance from developed countries. In addition to promoting interventions to reduce exposure, Emerging Market countries could focus efforts on continuing education for healthcare providers and public health professionals about exposure routes and prevention strategies. Authors also recommend further study and publication on pediatric metals exposures in Emerging Market countries as well as local dissemination of study findings and translation into action-based public health interventions with followup to evaluate the effects of interventions.

## Figures and Tables

**Table 1:** Criteria for inclusion of journal articles.

General category	Specific inclusion criteria
Chemical	Arsenic, cadmium, lead, or mercury
Country	Brazil, China, India, Indonesia, Mexico, Poland, Russia, South Korea, Taiwan, or Turkey
Age	≤18 years
Matrix analyzed	Blood or urine
Language	English only
Evidence of contamination or adverse health effects	Contains data on either levels of metal contamination in matrix analyzed or adverse health effects for population of interest

**Table:** (a)

Country	Ages	Specimen	Results*
Brazil	7–14 years	Urine	Median 3.60 versus 6.30, 6.40, 8.94 *μ*g/L (unexposed versus 3 exposed groups) *n* = 73 versus *n* = 129, 107, 89 [38]

China	Newborn	Cord blood	Mean 3.82 *μ*g/L, *n* = 142 [39]

India	Children	Urine	Range 23–4030 *μ*g/L, *n* = 298 [40]
	9–11 years	Urine	Range 570–2349 *μ*g/L, *n* = 7 [41]
	5–15 years	Urine	Mean 78 *μ*g/L (range 2–375), *n* = 349 [42]

Mexico	4–6 years	Urine	Mean 143.9 versus 24.8 *μ*g/L (exposed *n* = 7 versus unexposed *n* = 5) [43]
	6–8 years	Urine	Mean 58.1 *μ*g/L, *n* = 602 [44]
	6–11 years	Urine	Means 16.5 *μ*g/dL [45], 19.9 *μ*g/L [46], *n* = 50, 90
	6–11 years	Urine	Medians 143.0, 100.0, 115.0 *μ*g/L *n* = 21, 22, 22 [47]
	6–11 years	Urine	Medians 136.75, 106.25, 116.0 *μ*g/L *n* = 19, 21, 20 [48]
	6–12 years	Urine	Mean 22.35 *μ*g/g creatinine, *n* = 229 [49]

Poland	8–12 years	Urine	GMean 7.98 versus 5.99 *μ*g/g creatinine (*n* = 49 exposed versus *n* = 50 unexposed females) [37]
			GMean 8.74 versus 6.73 *μ*g/g creatinine (*n* = 44 exposed versus *n* = 34 unexposed males) [37]

**Table:** (b)

Country	Ages	Specimen	Results*
China	Newborn	Cord blood	Median 3.61 versus 1.25 *μ*g/L (*n* = 289 e-waste exposed versus *n* = 134 unexposed) [50]
			Mean 4.84 versus 2.81 *μ*g/L (*n* = 289 e-waste exposed versus *n* = 134 unexposed) [50]
	Newborn	Cord blood	Median 0.6 *μ*g/L (range 0.02–1.78), *n* = 109 [51]
	Newborn	Cord blood	GMean 0.36 *μ*g/L (range 0.02–1.48), *n* = 44 [52]

India	Newborn	Cord blood	GMean 0.6 *μ*g/L, *n* = 296 [53]

Mexico	6–11 years	Urine	Mean 4.7 *μ*g/L, *n* = 35 [45]
	6–12 years	Urine	GMean 0.78 *μ*g/L, *n* = 229 [49]

Poland	6-7 years	Whole blood	GMean 0.6 *μ*g/L, *n* = 183 [54]
	8–12 years	Whole blood	GMean 0.19 versus 0.08 *μ*g/L (*n* = 50 exposed versus *n* = 45 unexposed females) [37]
			GMean 0.19 versus 0.07 *μ*g/L (*n* = 42 exposed versus *n* = 35 unexposed males) [37]
	8–12 years	Urine	GMean 0.56 versus 0.45 *μ*g/g creatinine (*n* = 49 exposed versus 50 unexposed females) [37]
			GMean 0.68 versus 0.44 *μ*g/g creatinine (*n* = 44 exposed versus 35 unexposed males [37]

South Korea	4–10 years	Whole blood	GMean 1.51 *μ*g/L (range 0.05–6.00), *n* = 38 [55]
	4–10 years	Urine	GMean 1.33 *μ*g/L (range 0.02–5.25), *n* = 38 [55]
			GMean 1.69 *μ*g/g creatinine (range 0.43–3.92), *n* = 38 [55]

Taiwan	Newborn	Cord blood	Mean 0.67 *μ*g/L, median 0.33 *μ*g/L, *n* = 402 [56]

**Table:** (c)

Country	Ages	Specimen	Results
Brazil	6–8 years	Whole blood	Means 2.1 *μ*g/dL [57], 5.5 *μ*g/dL [58], *n* = 444, 65

China	Newborn	Cord blood	Mean 4.06 *μ*g/dL, *n* = 240 [59]
	Newborn	Cord blood	Means 3.6 *μ*g/dL [60], 11.33 *μ*g/dL [61], *n* = 110, 100
	Newborn	Cord blood	GMeans 5.35 versus 8.41 versus 6.0 *μ*g/dL (urban *n* = 24 versus rural *n* = 45 versus industrial residence *n* = 20) [62]
	Newborn	Cord blood	Median 4.36 *μ*g/dL (range 1.72–9.82) *n* = 109 [51]
	1–5 years	Whole blood	GMean 8.2 *μ*g/dL, *n* = 1117 [63]
	2 months–14 years	Whole blood	Means 16.38 versus 7.12 *μ*g/dL (*n* = 369 polluted versus *n* = 61 control area) [64]^†^
	<6 years	Whole blood	GMean 4.71 *μ*g/dL, *n* = 44,048 [65]
	6–12 years	Whole blood	Range 1.59–31.8 *μ*g/dL, *n* = 317 [66]
	4–13 years	Whole blood	GMean 6.71 *μ*g/dL, *n* = 307 [67]

India	Newborn	Cord blood	GMean 5.1 *μ*g/dL, *n* = 296 [53]
	Newborn	Cord blood	Means 11.4 versus 16.02 *μ*g/dL, *n* = 23 normal weight versus *n* = 24 growth retarded [68]
	Infants	Whole blood	Mean 10.15 *μ*g/dL (range 0.046–42.94), *n* = 200 [69]
	3–7 years	Whole blood	Mean 11.47 *μ*g/dL (range 2.6–40.5), *n* = 756 [70]
	3–12 years	Whole blood	Mean 4.23 versus 9.86 *μ*g/dL (*n* = 51 controls versus *n* = 17 aplastic anemia cases) [71]
	5–13 years	Whole blood	Mean 15.11 *μ*g/dL, *n* = 100 [72]
	15–18 years	Whole blood	Mean 9.96 *μ*g/dL (range 4.62–18.64), *n* = 39 [73]

Indonesia	6–12 years	Whole blood	GMean 8.6 *μ*g/dL (*n* = 397) [74]

Mexico	Newborn	Cord blood	Means, 2.7 *μ*g/dL [75], 6.2 *μ*g/dL [76], 8.1 *μ*g/dL [77], 6.6 *μ*g/dL [78], 6.7 *μ*g/dL [79], 6.6 *μ*g/dL [80], 5.49 *μ*g/dL [81], *n* = 226, 146, 617, 424, 197, 364, 294
	1 month	Whole blood	Mean 5.5 *μ*g/dL (range 1–23.1), *n* = 222 [82]
	12 month	Whole blood	Means 4.6 *μ*g/dL [81], 7.2 *μ*g/dL [79], *n* = 294,197
	1 year	Whole blood	GMean 8.4 *μ*g/dL, *n* = 302 [83]
	2 years	Whole blood	GMean 10.1 *μ*g/dL, *n* = 264 [83]
	2 years	Whole blood	Means 4.8 *μ*g/dL [76], 5.78 *μ*g/dL [81], 8.2 *μ*g/dL [80], 8.4 *μ*g/dL [79], *n* = 146, 752, 283, 179
	3 years	Whole blood	Mean 8.4 *μ*g/dL, *n* = 206 [80]
	4 years	Whole blood	Mean 8.2 *μ*g/dL, *n* = 227 [80]
	6–8 years	Whole blood	Median 10.2 *μ*g/dL (range 1.9–43.8) *n* = 598 [84]
	6–8 years	Whole blood	Mean 11.5 *μ*g/dL, *n* = 21 [44]
	6–11 years	Whole blood	Means 4.6 *μ*g/dL [45], 9.4 *μ*g/dL [49], *n* = 229, 50
	6–11 years	Whole blood	Median by group, with increasing proximity to source: 4.6, 9.5, 28.6 *μ*g/dL, *n* = 21, 22, 22 [48]
	6–11 years	Whole blood	Median by group with increasing proximity to source: 7.02, 20.6, 30.38 *μ*g/dL, *n* = 21,21,23 [47]
	6–12 years	Whole blood	GMeans 10.5, 11.2, 12.4 *μ*g/dL, *n* = 12,22,24 [85]

Poland	Newborn	Cord blood	Median 1.23 *μ*g/dL (range 0.44–6.90), *n* = 444 [86]
	Newborn	Cord blood	Mean 1.42 *μ*g/dL, *n* = 452 [87]
	5–14 years	Whole blood	Mean 7.69 *μ*g/dL, median 6.77 *μ*g/dL (range 2.7–23), *n* = 74 [88]
	6-7 years	Whole blood	GMean 4.2 *μ*g/dL, *n* = 202 [54]
	7–15 years	Whole blood	Mean 7.3 *μ*g/dL, median 6.6 *μ*g/dL, *n* = 272 [89]^†^
	8–12 years	Whole blood	GMean 5.72 versus 3.42 *μ*g/dL (*n* = 47 exposed versus *n* = 50 unexposed females) [37]
			GMean 6.51 versus 3.81 *μ*g/dL (*n* = 42 exposed versus *n* = 35 unexposed males) [37]

South Korea	4–10 years	Whole blood	GMean 3.80 *μ*g/dL (range 1.30–9.95) *n* = 38 [55]
	10–15 years	Whole blood	Mean 4.3 versus 6.9 *μ*g/dL (*n* = 167 controls versus *n* = 39 anemia) [90]

Taiwan	Newborn	Cord blood	Means 7.8 versus 3.28 versus 2.35 *μ*g/dL in 1985–87 versus 1990–92 versus 2001-02 [91]
	Newborn	Cord blood	Mean 1.29 *μ*g/dL, *n* = 308 [92]
	Newborn	Cord blood	GMeans 1.26 *μ*g/dL [93], 1.30 *μ*g/dL [94], *n* = 1526, 430
	2-3 years	Whole blood	GMean 2.48 *μ*g/dL, *n* = 430 [94]
	5-6 years	Whole blood	GMean 2.49 *μ*g/dL, *n* = 430 [94]
	8-9 years	Whole blood	GMean 1.97 *μ*g/dL, *n* = 430 [94]
	7–11 years	Whole blood	Means 1.60 versus 7.79 *μ*g/dL (*n* = 71 controls versus *n* = 79 exposed males) [95]
	8–12 years	Whole blood	Means 3.45 versus 5.23 versus 8.80 *μ*g/dL, *n* = 100 unexposed versus *n* = 30 and 34 exposed [96]

Turkey	Newborn	Cord blood	Mean 1.65 *μ*g/dL, *n* = 120 [97]
		Whole blood	Mean 3.56 *μ*g/dL, *n* = 180 [97]
	15–19 years	Whole blood	Mean 7.8 versus 1.6 *μ*g/dL (*n* = 79 exposed versus *n* = 71 unexposed) [98]

**Table:** (d)

Country	Ages	Specimen	Results
Brazil	Newborn	Cord blood	GMean 9.63 *μ*g/L, *n* = 61 [99]
	Newborn	Cord blood	Mean 16.68 *μ*g/L (range 0.35–135.04), *n* = 1510 [100]

China	Newborn	Cord blood	GMean 5.58 *μ*g/L, *n* = 384 [101]
	Newborn	Cord blood	Mean 7.0 *μ*g/L (range 2.28–39.72), *n* = 110 [60]

Indonesia	9–17 years	Urine	Medians 0.32 versus 7.06 *μ*g/g creatinine (control *n* = 50 versus occupationally exposed *n* = 80) [102]
		Whole blood	Medians 3.47 versus 7.75 *μ*g/L (control *n* = 50 versus occupationally exposed *n* = 80) [102]

Mexico	6–11 yr	Urine	GMean 4.2 *μ*g/g creatinine, *n* = 23 [103]
	6–12 yr	Urine	Mean 0.7 *μ*g/L, *n* = 50 [45]

Poland	Newborn	Cord blood	Mean 1.06 *μ*g/L, *n* = 313 [104]
	Newborn	Cord blood	GMean 0.88 *μ*g/L (range 0.10–5.00) *n* = 233 [105]

*Results are expressed as they were provided: arithmetic mean (mean), geometric mean (Gmean), or median value.

^†^Study of occupational take-home exposure.

**Table 3:** Reference values for the United States population (in *μ*g/L), NHANES 2005-2006 [110].

Metal	Ages (years)	Specimen^†^	Geometric mean (95% CI)	50th percentile (95% CI)	95th percentile (95% CI)
Arsenic	6–11	U	7.19 (5.81–8.90)	6.96 (5.32–8.88)	34.1 (19.6–58.5)
		UCC	8.88 (7.05–11.2)	7.87 (6.19–9.42)	45.4 (22.9–80.9)
	12–19	U	8.19 (6.87–9.77)	7.92 (6.37–9.50)	41.9 (32.7–48.0)
		UCC	6.30 (5.56–7.14)	5.19 (4.80–6.19)	28.0 (21.9–33.2)

Cadmium	1–5	WB	NA*	NA	0.230 (0.210–0.250)
	6–11	WB	NA	NA	0.260 (0.230–0.280)
	12–19	WB	NA	NA	0.960 (0.820–1.08)
	6–11	U	0.066 (0.056–0.078)	0.060 (0.050–0.080)	0.240 (0.160–0.290)
		UCC	0.081 (0.072–0.092)	0.080 (0.070–0.090)	0.200 (0.180–0.240)
	12–19	U	0.099 (0.090–0.109)	0.110 (0.100–0.120)	0.310 (0.250–0.430)
		UCC	0.076 (0.071–0.081)	0.080 (0.070–0.090)	0.210 (0.160–0.240)

Lead^‡^	1–5	WB	1.46 (1.36–1.57)	1.43 (1.34–1.55)	3.80 (3.49–4.54)
	6–11	WB	1.02 (0.948–1.10)	0.970 (0.890–1.01)	3.00 (2.26–3.81)
	12–19	WB	0.797 (0.746–0.852)	0.740 (0.690–0.790)	2.23 (1.98–2.46)

Mercury	1–5	WB	NA	NA	1.43 (1.25–1.59)
	6–11	WB	NA	0.410 (0.330–0.460)	2.34 (1.53–3.42)
	12–19	WB	0.513 (0.461–0.570)	0.460 (0.390–0.530)	2.41 (2.12–2.90)
	6–11	U	0.333 (0.267–0.416)	0.320 (0.250–0.390)	2.18 (1.28–3.40)
		UCC	0.411 (0.323–0.524)	0.390 (0.290–0.500)	2.55 (1.38–3.50)
	12–19	U	0.372 (0.286–0.486)	0.350 (0.270–0.470)	2.59 (1.40–4.45)
		UCC	0.286 (0.230–0.356)	0.260 (0.200–0.320)	1.76 (1.11–2.67)

^†^U: urine; UCC: urine, creatinine corrected (in *μ*g/g of creatinine); WB: whole blood.

^‡^Lead measurements reported in *μ*g/dL.

*NA: not available or not calculated due to low detection frequency.

**Table 4:** Conclusions and recommendations.

Conclusions	
(i) There is evidence of pediatric exposure to As, Cd, Pb, and Hg in Emerging Market countries, often as a result of industrial development activities.	
(ii) There is indication that children's health is being affected as a result of these exposures.	
(iii) Limited studies in the peer-reviewed literature document the extent of metals exposure and health consequences in Emerging Market countries.	
(iv) Country-specific reference values are largely unavailable in Emerging Market countries making interpretation of exposure assessment difficult.	
(v) There is incomplete knowledge of the public health impact of exposure to As, Cd, Pb, and Hg in Emerging Market countries.	

Recommendations	

(i) Authors recommend further study and publication on pediatric metal exposures and interventions to decrease exposures in Emerging Market countries.	
(ii) Authors recommend development of country-specific reference values for these metals.	
(iii) Authors recommend ensuring local dissemination of study findings and translation into action-based public health interventions with followup to evaluate the effects of interventions.	
(iv) Authors recommend continuing education for healthcare providers and public health professionals about exposure routes and prevention strategies.	
